# Perceived Social Status and Oral Health Among Medicaid Insured Adults in Iowa

**DOI:** 10.1089/heq.2023.0227

**Published:** 2024-09-23

**Authors:** Jennifer M.C. Sukalski, Natoshia M. Askelson, Julie C. Reynolds, Peter C. Damiano, Wei Shi, Xian Jin Xie, Susan C. McKernan

**Affiliations:** ^1^ Department of Preventive and Community Dentistry, University of Iowa College of Dentistry, Iowa City, Iowa, USA.; ^2^ Department of Community and Behavioral Health, University of Iowa College of Public Health, Iowa City, Iowa, USA.; ^3^ Division of Biostatistics and Computational Biology, Iowa Institute for Oral Health Research, University of Iowa College of Dentistry, Iowa City, Iowa, USA.; ^4^ Department of Public and Primary Oral Healthcare, University of Minnesota School of Dentistry, Minneapolis, Minnesota, USA.

**Keywords:** perceived social status, oral health, social class, health inequalities, health status, health surveys

## Abstract

**Purpose:**

Perceived social status (PSS), a measure of social status, reflects cumulative lifetime effects of an individual’s relative social status based on resources and lived experiences. PSS is hypothesized to better capture social status compared to traditional measures of socioeconomic status (SES) (i.e., education, occupation, and income). Although recognized as a predictor of health-related morbidity and mortality, limited research has explored PSS and oral health. This study investigated PSS as a predictor of self-reported oral health among low-income adults.

**Methods:**

In spring 2018, a survey was administered to a random sample of low-income adults in the state of Iowa with public dental insurance (*N* = 18,000). Respondents were asked about PSS, oral health status, and demographics. Multivariable linear regression models examined PSS as a predictor of self-reported oral health and compared the predictive power of PSS and SES indicators.

**Results:**

The final adjusted sample size was 2,331. The mean PSS (range 1-10) was 5.3 (standard deviation 2.0). A significant positive association was noted between PSS (*ß* = 0.16, *p* < 0.0001) and self-reported oral health status when controlling for demographics. Furthermore, PSS accounted for an additional 3% of variance when controlling for demographic and SES indicators.

**Conclusions:**

PSS was associated with self-reported oral health status after adjusting for SES indicators, which reflects the importance of exploring the impact of individuals’ perceptions of their social status in addition to objective measures of SES. Results suggest the need for future dental research to explore cumulative effects of lived experiences on current oral health status.

## Introduction

Perceived social status (PSS) denotes how individuals view their position within the social hierarchy relative to others, accounting for variation in lived experiences.^1,2^ PSS differs from traditional measures of socioeconomic status (SES)—education, occupation, and income—which are objective and typically measured at a single point in time.^3^ Although SES measures are valid and reliable at the time of measurement, this approach has limitations.^3,4^ They apply fixed criteria of attainment, failing to account for variability in the significance and value that an individual ascribes to their lived experiences.^4^

Measures of SES are contextually inadequate, ignoring factors such as the environment born and lived in, access to quality education, financial stability, and social networks.^4^ A measure of education, for example, may only capture years completed or degrees obtained. It omits access to quality education, available resources, or perceived prestige. To one individual, education from a 2-year college may be more significant to their prospective life course than someone with a more perceived prestigious education. A 2-year degree could change one’s financial situation, employment opportunities, and forever impact their self-perception. This ascribed value may be intensified for individuals from a social environment in which postsecondary educational attainment rates are low. Similarly, occupation may fail to account for unequal employment opportunities or fluctuation in income. Someone from a more affluent community may rate themselves as having lower PSS if they perceive themselves as having declined in status based on their experiences—loss of job or income. Thus, the objective measures of SES do not account for the individual’s perception of their relative social status.

To account for the cumulative effects of lived experiences, the MacArthur Research Network on SES and Health developed a validated measure of perceived social status (PSS).^5–7^ PSS was designed to better capture ones’ sense of status in the social hierarchy.^6^ The PSS survey item asks individuals to consider education, occupation, and income of others in the United States and to select their own relative position on the “social ladder.”^6^ Thus, the PSS measure is a self-anchoring item, whereby individuals define the values of the scale based on their perceptions, goals, and values.^8^ PSS allows for the evaluation of the person holistically, considering their cumulative life experiences in a way that objective SES items are unable to account for.^9,10^

PSS has been found to be a predictor of physical^10,11^ and mental health,^10–13^ stress,^11^ self-reported health,^11^^,^^12^^,^^14^ and even mortality.^15^^,^^16^ An analysis of British civil servants found that PSS was a greater predictor of poor health compared to SES. Results showed that lower PSS was related to higher rates of age-adjusted morbidity of angina, diabetes, depression, and lower overall health (*p* < 0.0001). When both SES measures and PSS were accounted for in analyses, only PSS remained significantly associated with adverse health outcomes.^12^ A study of U.S. pregnant women found that PSS was more strongly associated with self-reported health than SES (*p* < 0.0001).^2^ These studies demonstrated that PSS can be more predictive of health outcomes than SES measures, and that SES may not account for all aspects of inequality.^15^

Few studies have evaluated the relationship between PSS and oral health. Studies examining PSS and oral health have documented associations between lower PSS and missing teeth, decreased chewing capacity, and lower overall self-reported oral health.^16^ Higher PSS scores have also been linked with fewer oral health functional limitations and disabilities,^17^ increased likelihood of having a dental home,^18^ and increased likelihood of utilizing dental services.^19^ Although studies suggest a relationship between PSS and oral health, further investigation is necessary to better understand the multidimensional impact of social status on oral health outcomes in low-income populations with restricted resources who face barriers to maintaining good oral health.

The aims of this study were 1) to explore the association between PSS and oral health status among a low-income adult population in the state of Iowa and 2) to evaluate whether PSS provides more predictive power than objective SES measures regarding oral health status. We hypothesized that PSS would have a positive association with oral health and hypothesize that PSS would provide more additional predictive power related to oral health status than objective measures of SES as it contextually reflects the accumulation of life experiences.

## Methods

In spring 2018, paper surveys were mailed to a simple random sample of 19-64-year-old Medicaid enrollees (*N* = 18,000). Members were also able to complete the survey online. Postcard reminders were sent 2 weeks after the initial mailing. A second survey was mailed to nonrespondents 2 weeks later. As an incentive, all recipients received a $2 bill in the first survey mailing. Those who returned the survey within 2 weeks were entered into a drawing for one of ten $100 retail store gift cards.

**FIG. 1.**
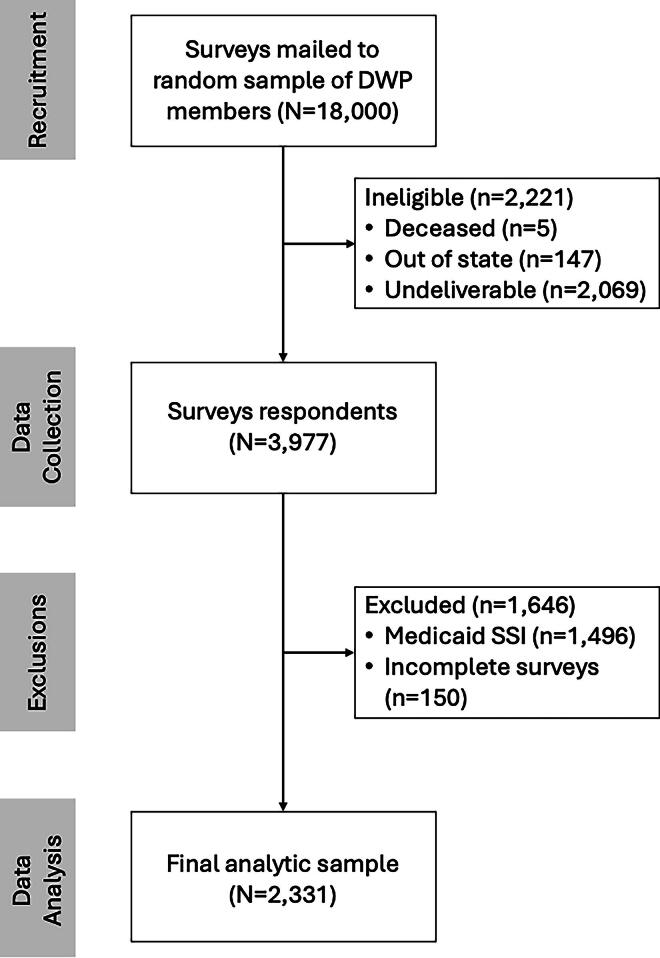
Flowchart depicting analytical sample selection.

The sampling frame for the survey included members who had been enrolled for at least 6 months, and those enrolled through either Iowa’s Medicaid dental program or the Dental Wellness Plan. The sample frame excluded pregnant enrollees, and only one person per household could be selected. Based on Medicaid eligibility requirements, all individuals had incomes at 0%–138% of the federal poverty level (FPL) (Figure 1).^20^

The University of Iowa Human Subjects Office determined that this project did not meet the regulatory definition of human subject research under a waiver approved by the Secretary, U.S. Department of Health and Human Services.

### Variables

#### Oral health status

The dependent variable was self-reported oral health status. Respondents were asked: “*In general, how would you rate the overall condition of your teeth and gums?*” with a 5-point Likert response option ranging from Poor (1) to Excellent (5) and treated as continuous in the multivariable analyses.

#### PSS

PSS, the independent variable of interest, was a modified version of the MacArthur ladder (Supplementary Appendix SA1).^20^ Respondents were asked “*Please think of how you see yourself compared to other people in society. One a scale of 1 to 10, where 1 are people who are the worst off and 10 are people who are the best off, where would you place yourself?*” Modifications to the survey item were made to maintain consistency with the rest of the survey. Modifications included removal of the ladder graphic, referring to response options as a “scale” rather than “ladder,” and having respondents fill in a box associated with their perceived location on the scale rather than an “x” on a ladder rung. Respondents were given numeric response options that ranged from 1 through 10. Option 1 included the description “Worst off: least education, least money, worst jobs or no jobs.” Option 10 indicated the top of the ladder and included the description “Best off: most education, most money, best jobs.”

#### Demographic variables

Demographic variables were selected based on supporting literature documenting an association with oral health, including age, sex, race/ethnicity, marital status, and rurality. Rurality was based on postal code (ZIP) data and categorized by the Rural-Urban Commuting Area classification system into three levels: “*Urban*,” “*Large rural*,” and “*Small rural*.”^21^

#### SES measures

Three SES measures were examined: educational attainment, employment status, and income. Respondents reported their highest level of education achieved, and current level of employment at the time of the survey. Income was obtained from Medicaid enrollment data, expressed as a percentage of the U.S. poverty level with values ranging from 0% to 138% of the U.S. FPL.

### Statistical analyses

Descriptive statistics and correlation analyses were performed to assess associations between oral health status and the independent variables. A series of four multiple linear regression models were completed to examine PSS as a predictor of oral health status and compare PSS against SES measures. Variables were entered as blocks corresponding to (1) demographic characteristics, (2) demographic characteristics plus SES measures, (3) demographic characteristics plus PSS, and (4) demographic characteristics, SES measures, and PSS. Multicollinearity was evaluated using tolerance (range: 0.76–0.99) and variance inflation factor (range: 1.04–1.31), which yielded acceptable values.

Explanatory power of SES measures relative to PSS was evaluated by comparing beta coefficients and the statistical significance of predictors across models. Differences in the variance of oral health status with the addition of PSS to the model were examined by changes in *R*^2^ and in the *F*-test of overall model significance. *F*-tests indicated whether the linear regression model provided a better fit to the data than an intercept-only model.

All analyses were completed using SPSS Version 27 (Armonk, NY, USA). A significance level of *p* ≤ 0.05 was used for all hypothesis testing. Ordinal logistic regression and binary logistic regression models were also generated to evaluate robustness of the linear models. In addition, to assess for a nonlinear relationship between age and the outcome of interest, age-squared was included in the final linear regression models and presented in Supplementary Appendix SA2.

## Results

The adjusted sample size was 15,779 after excluding undeliverables. A total of 3,977 samples completed the survey for an adjusted response rate of 25%. The final sample size was 2,331 after removing individuals who did not meet the inclusion criteria—removal of individuals enrolled in Supplemental Security Income and Disability Medicaid (*n* = 1,496) and surveys with missing data (*n* = 150) (Figure 1).

**FIG. 2.**
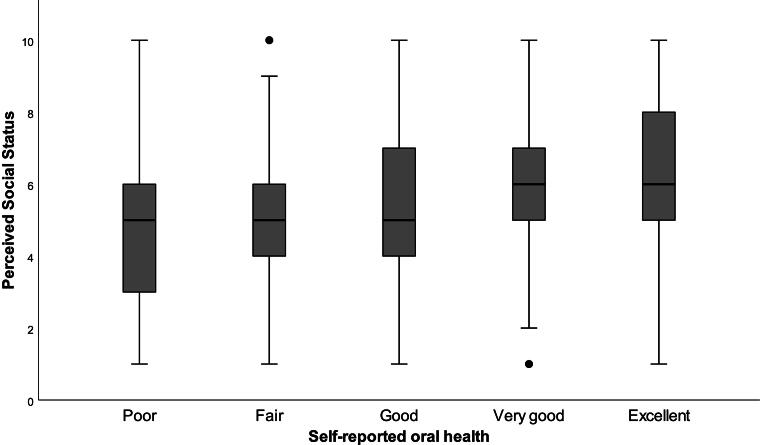
Box and whisker plot of the distribution of perceived social status by self-reported oral health among Iowa Medicaid Beneficiaries (*N* = 2,331).

Descriptive statistics for all variables are presented in Table 1. Mean PSS was 5.3 (standard deviation 2.0, range 1–10). Figure 2 shows the distribution of PSS and oral health status among respondents. The median PSS score for individuals who reported poor, fair, and good oral health was 5.0, whereas individuals who reported very good or excellent health respondents had median PSS of 6.0. Although median values of PSS were consistent across oral health categories, the interquartile ranges for PSS scores varied.

**Table 1.** Distribution of Characteristics of Responding Iowa Medicaid Beneficiaries, 2018 (*N* = 2,331)

	*N* (%) or mean (SD)
Self-reported oral health	
Excellent	171 (7.3)
Very good	496 (21.3)
Good	776 (33.3)
Fair	515 (22.1)
Poor	373 (16.0)
Perceived Social Status (PSS) mean (SD)	5.3 (SD 2.0)
Age years, mean (SD)	41.06 (SD 12.2)
Sex	
Male	675 (29.0)
Female	1,656 (71.0)
Race/Ethnicity	
White	1,893 (81.2)
Black	138 (5.9)
Hispanic/Latino	88 (3.8)
Asian	50 (2.1)
Other race or multiracial	162 (6.9)
Marital status	
Married	869 (37.3)
Divorced	522 (22.4)
Widowed	64 (2.7)
Separated	106 (4.5)
Single	770 (33.0)
Rurality	
Urban	1,256 (53.9)
Large rural	427 (18.3)
Rural	648 (27.8)
Educational attainment	
8th grade or less	49 (2.1)
Some high school	214 (9.2)
High school graduate or GED	875 (37.5)
Some college or 2-year degree	911 (39.1)
4-year college graduate	204 (8.8)
More than 4-year college degree	78 (3.3)
Employment status	
Employed full-time	529 (22.7)
Employed part-time	591 (25.4)
Out of work for less than 1 year	192 (8.2)
Out of work for 1 year or more	454 (19.5)
Homemaker	340 (14.6)
Student	153 (6.6)
Retired	72 (3.1)
Percent FPL, mean (SD)	37.77 (SD 45.1)

FPL, federal poverty level

Table 2 shows correlations between PSS, SES measures, and oral health. PSS had a small positive correlation with percent FPL (*r* = 0.16), educational attainment (*r* = 0.20), and oral health (*r* = 0.28). Oral health was more strongly correlated with PSS (*r* = 0.28) than with educational attainment (*r* = 0.22) or percent FPL (*r* = 0.08). Educational attainment and percent FPL had a negligible positive correlation (*r* = 0.05).

**Table 2.** Pearson Correlations between Perceived Social Status and Socioeconomic Status Measures

	Self-reported oral health	Perceived social status	Educational attainment	Percent FPL
Self-reported oral health	1.00			
Perceived social status	0.28^**^	1.00		
Educational attainment	0.22^**^	0.20^**^	1.00	
Percent FPL	0.08^**^	0.16^**^	0.05^*^	1.00

^*^ *p* < 0.05.

^**^ *p* < 0.01.

FPL, federal poverty level.

Results of the multiple linear regression analyses are shown in Table 3. The effects of age were statistically significant and consistent across all models; as age increased, oral health decreased. Model 1 contained demographic variables only. Sex and rurality were not significantly associated with oral health status. In model 1, demographic variables accounted for 2% of variation in oral health status, which was a significant increase in variation relative to the null model (*R*^2^ = 0.02, *F*-change = 3.98, *p* < 0.001).

**Table 3.** Multiple Linear Regression Analyses Predicting Higher Self-Reported Oral Health Status (*n* = 2,331)

	Model 1 ß (95% CI)	Model 2 ß (95% CI)	Model 3 ß (95% CI)	Model 4 ß (95% CI)
Demographics				
Age (years)	−0.01 (−0.01, −0.003)^***^	−0.01 (−0.01, −0.002)^**^	−0.01 (−0.01, −0.003)^**^	−0.01 (−0.01, −0.002)^**^
Sex Female Male	0.06 (−0.05, 0.16) Ref	0.02 (−0.09, 0.12) Ref	0.04 (−0.07, 0.14) Ref	0.01 (−0.10, 0.11) Ref
Race/Ethnicity Black Hispanic/Latino Asian Other or multiple race White	0.22 (0.01, 0.42)^*^ 0.21 (−0.04, 0.45) 0.22 (−0.12, 0.54) 0.08 (−0.12, 0.26) Ref	0.31 (0.15, 0.51)^**^ 0.22 (−0.02, 0.46) 0.18 (−0.14, 0.51) 0.13 (−0.05, 0.31) Ref	0.11 (−0.09, 0.30) 0.11 (−0.12, 0.35) 0.20 (−0.13, 0.50) 0.09 (−0.09, 0.27) Ref	0.22 (0.02, 0.41)^*^ 0.15 (−0.09, 0.38) 0.16 (−0.16, 0.47) 0.12 (−0.05, 0.30) Ref
Marital status Divorced Widowed Separated Single Married	−0.08 (−0.21, 0.05) −0.06 (−0.36, 0.24) −0.38 (−0.61, −0.15)^**^ 0.02 (−0.09, 0.14) Ref	−0.10 (−0.23, 0.03) 0.01 (−0.27, 0.30) −0.24 (−0.47, −0.02)^*^ 0.02 (−0.11, 0.11) Ref	−0.03 (−0.16, 0.09) 0.01 (−0.28, 0.29) −0.27 (−0.49, −0.04)^**^ 0.05 (−0.06, 0.16) Ref	−0.06 (−0.18, 0.07) 0.04 (−0.24, 0.32) −0.20 (−0.42, 0.03) 0.02 (−0.09, 0.13) Ref
Rurality Small rural Large rural Urban	0.02 (−0.09, 0.14) −0.07 (−0.20, 0.06) Ref	0.05 (−0.057, 0.16) −0.05 (−0.17, 0.07) Ref	0.04 (−0.07, 0.15) −0.05 (−0.17, 0.08) Ref	0.06 (−0.04, 0.17) −0.03 (−0.15, 0.09) Ref
SES measures				
Educational attainment 8^th^ grade or less Some high school High school graduate or GED Some college or 2-year degree 4-year college graduate More than 4-year college		−0.87 (−1.28, 0.47)^***^ −0.97 (−1.26, −0.68)^***^ −0.81 (−1.07, −0.55)^***^ −0.54 (−0.80, −0.28)^***^ −0.12 (−0.49, 0.09) Ref		−0.70 (−1.06, −0.25)^**^ −0.82 (−1.12, −0.53)^***^ −0.68 (−0.94, −4.29)^***^ −0.46 (−0.71, −0.20)^***^ −0.16 (−0.45, 0.12) Ref
Employment status Employed part-time Out of work for <1 year Out of work for ≥1 year Homemaker Student Retired Employed full-time		−0.06 (−0.19, 0.07) −0.31 (−0.50, −0.12)^**^ −0.18 (−0.33, −0.03)^*^ −0.02 (−0.19, 0.14) 0.47 (0.26, 0.67)^***^ 0.13 (−0.16, 0.41) Ref		−0.02 (−0.15, 0.11) −0.20 (−0.38, −0.01)^*^ −0.02 (−0.17, 0.13) 0.05 (−0.11, 0.21) 0.45 (0.25, 0.66)^***^ 0.09 (−0.19, 0.37) Ref
Percent poverty		0.002 (0.001, 0.003)^**^		0.001 (0.000, 0.003)^**^
Perceived Social Status				
PSS			0.16 (0.14, 0.18)^***^	0.124 (0.10, 0.15)^***^
Model and change statistics *R*^2^ Δ*R*^2^ F change	0.02 0.02 3.98^***^	0.10 0.08 17.67^***^	0.09 0.07 178.92^***^	0.13 0.03 100.44^***^

Dependent variable: self-reported oral health status (excellent = 5, very good = 4, good = 3, fair = 2, poor = 1).

^*^ *p* < 0.05.

^**^ *p* < 0.01.

^***^ *p* < 0.001.

CI, confidence interval; GED, General Educational Development

SES measures were added in model 2; all SES measures were significantly associated with oral health status. Respondents with educational attainment levels of some college or 2-year degree (*ß* = −0.87, *p* < 0.0001), high school graduate or GED (*ß* = −0.97, *p* < 0.0001), some high school (*ß* = −0.81, *p* < 0.0001), and eight grade or less (*ß* = −0.54, *p* < 0.0001) had significantly lower oral health compared to those with more than 4 years of college. Regarding employment status, respondents who were out of work for less than 1 year (*ß* = −0.31, *p* < 0.05) or more than 1 year (*ß* = −0.18, *p* < 0.01) had lower oral health compared to full-time employment. Being a student (*ß* = 0.47, *p* < 0.001) and percent FPL (*ß* = 0.002, *p* < 0.01) were positively associated with oral health status. The model explained 10% of variation in oral health status, and the change in *R*^2^ from model 1 to model 2 was 0.08, indicating an 8% increase in explained variation (*p* < 0.001).

Model 3 tested the association between PSS and oral health status, controlling for demographics. Results demonstrated a significant positive association between PSS (*ß* = 0.16, *p* < 0.0001) and oral health, and explained 9% of variation in the dependent variable. The full model (model 4), all SES-measures, and PSS remained significant. The addition of PSS accounted for an additional 3% of the variance in oral health relative to model 2 (*p* < 0.001).

## Discussion

PSS was associated with oral health status after adjusting for objective SES measures. Results support our first hypothesis: individuals with lower PSS were more likely to report lower oral health. The second hypothesis was supported; SES indictors explained only slightly more variation in oral health status than PSS and PSS accounted for an additional 3% of variance in oral health beyond SES measures—a 33% increase in *R*^2^ compared to the model without PSS. This is consistent with the previous research that found that PSS accounted for an additional 2.3% of variance in self-reported oral health while controlling for SES^16^ and 6% in overall health.^2^

Our findings suggest that although SES measures are significant predictors of oral health, PSS accounts for an additional aspect of SES. This increase in *R*^2^ taps into the effects of cumulative life events that SES does not account for. This is consistent with previous studies that have documented associations between PSS and health outcomes, such as oral health,^16–18,22^ self-reported general health,^2,10–13^ mental health,^10–13^ hypertension,^10^ and diabetes.^10,12^

PSS is hypothesized to better capture social status compared to SES measures often used in health disparities research, as it contextually reflects lived experiences.^11^ Research on disparities in health often only uses education, income, and occupation in conceptual frameworks and analyses; at times only using one measure as a proxy for SES.^3,22,23^ However, our findings (Table 2) and previous research indicates that these correlations between education and income are not strong enough to recommend this approach.^3^

Furthermore, incomes can vary at similar educational levels, especially when considering contextual factors. Although individuals in our study sample were all categorized broadly as low-income based on Medicaid eligibility requirements, individuals’ educations, employment statuses, and income relative to the FPL varied substantially. In addition, it is important to highlight the complexities surrounding employment status that the SES variable fails to consider. The experience of employment status varies significantly, as would the stressful emotions or disappointment with how employment status was obtained. Unemployment, for example, is not a single variable but rather is a complicated spectrum of voluntary and involuntary situations, such as being unemployed but wanting or needing employment, being unemployed but not wanting employment, a student, a homemaker, or being retired. The control of an individual’s ability to obtain employment influences life experiences. PSS therefore allows for the individual to consider the social context surrounding employment status.

Financial hardships may explain the association between oral health and PSS by imposing barriers to maintaining good oral health. However, we were not able to measure other aspects of financial hardship, such as poverty, material hardship, or subjective financial stress, beyond income as a proportion of the poverty level. Individuals undergoing financial hardships may be less likely to maintain good oral health and if individuals with lower levels of oral health are unable to access necessary dental care, they may be more likely to perceive themselves to have lower PSS.

Individuals with lower PSS may experience higher levels of chronic stress, anxiety, and other effects of hardships that could directly or indirectly influence oral health.^10–12^ Chronic stressors have long-lasting negative impacts on systemic health, including increased blood pressure and inflammatory markers.^24^ The consequences of chronic stress on oral health have been noted in the literature, as chronic stress has shown to increase the severity of periodontal disease and contribute to poor outcomes after periodontal treatment.^25^ PSS in adults may capture information about long-term exposure to adversities or available resources not fully captured by objective SES measures.

The primary limitation to this study was the cross-sectional design. We are unable to identify causal relationships based on cross-sectional data. Nonresponse error may pose a limitation. Analyses of nonresponse revealed that respondents were more likely to be older, White, and female compared with nonrespondents. This study may not be generalizable to other low-income populations in other states as it was conducted in a single U.S. state. The low response rate can also be considered a limitation; however, low response rates are common in mailed surveys of Medicaid members^26^ (typically 20–30% range),^27^ and low response rates may not indicate response bias.^28^

This study offers unique insights into the PSS of a low-income adult population. It highlights the importance of exploring oral health disparities using a more holistic approach and adds to the small body of work that has focused on PSS and oral health. Our findings suggest that PSS may be of importance to capture oral health outcomes in low-income adults. This can improve our understanding of the mechanisms underlying overall oral health. Better understanding of these cumulative effects can be used to help target public health resources.

Assessing PSS can provide valuable insights into disparities in oral health outcomes. This item could be used on local, state, or national surveys. Potential uses of the PSS item might include it to assess how much support someone might need to get dental care and be able to obtain routine dental care, which in turn can impact oral health outcomes. Previous research has shown that PSS has been associated with dental utilization,^19^ and along with this study demonstrating that PSS is associated with self-reported oral health status—assessing that PSS may indicate vulnerable populations to target interventions to improve oral health outcomes. Additional research is required to further understand the dynamics of PSS and mechanisms linking PSS to oral health outcomes.

## Conclusions

PSS was found to be associated with oral health status after adjusting for SES, indicating that SES is not capturing contextual factors—such as lived experiences and the impact those experiences have on self-perception of social status. Thus, reflecting the importance of exploring the impact of individuals’ perceptions of their social status in addition to objective SES measures. This suggests the need to explore these constructs beyond the SES and evaluate how lived experiences impacts health outcomes.

## Supplementary Material

Supplementary Appendix SA1

Supplementary Appendix SA2

## Abbreviations Used

FPL: Federal Poverty Level
GED: General Educational Development
PSS: Perceived Social Status
SES: Socioeconomic Status
