# Supplementary material for: Perceived Social Status and Oral Health Among Medicaid Insured Adults in Iowa
Source: Health Equity. 2024 Sep 23;8(1):667–75. doi: 10.1089/heq.2023.0227 (PMC11464866; doi:10.1089/heq.2023.0227)
Supplement: Supplementary Appendix SA1 [file heq.2023.0227_pss_modified_heq_appendixa.pdf]

Please think of how you see yourself compared to other people in society. On a scale of 1 to 10, where 1 are people who are the worst off and 10 are people who are the best off, where would you place yourself?

- 10 ☐ 10 **Best off:** most education, most money, best jobs
- 09 ☐ 9
- 08 ☐ 8
- 07 ☐ 7
- 06 ☐ 6
- 05 ☐ 5
- 04 ☐ 4
- 03 ☐ 3
- 02 ☐ 2
- 01 ☐ 1 **Worst off**: least education, least money, worst jobs or no jobs
